# The acetabulum in Perthes’ disease: a prospective study of 123 children

**DOI:** 10.1007/s11832-014-0617-9

**Published:** 2014-11-20

**Authors:** S. Huhnstock, S. Svenningsen, A. H. Pripp, T. Terjesen, O. Wiig

**Affiliations:** 1 Department of Pediatric Orthopaedic Surgery, Oslo University Hospital, Rikshospitalet, 0027 Oslo, Norway; 2 Orthopaedic Department, Sørlandet Hospital HF, Sykehusveien 1, 4838 Arendal, Norway; 3 Department of Biostatistics, Epidemiology and Health, Oslo University Hospital, 0027 Oslo, Norway

**Keywords:** Legg–Calvé–Perthes’ disease, Acetabulum, Hip, Secondary hip dysplasia

## Abstract

**Purpose:**

We assessed the radiographic changes of the acetabulum during the course of Perthes’ disease and investigated whether they were associated with femoral head sphericity 5 years after diagnosis.

**Methods:**

We studied 123 children with unilateral Perthes’ disease, femoral head necrosis more than 50 % and age at diagnosis 6 years or older. Pelvic radiographs were taken at onset, 1 year and 5 years after diagnosis. Sharp’s angle, acetabular depth-to-width ratio (ADR) and lateral acetabular inclination were measured.

**Results:**

Compared to the unaffected hips, the Perthes’ hips developed significantly higher Sharp’s angles (*p* < 0.001) and a higher proportion with an upward-sloping lateral acetabular margin (Perthes’ hips: 49 %, unaffected hips 1 %). The mean ADR values were significantly lower on the affected side at all stages (*p* < 0.001). ADR values at diagnosis were associated with a more spherical femoral head at the 5-year follow-up [odds ratio (OR) 1.012, 95 % confidence interval (CI) 1.002–1.022, *p* = 0.016]. None of the other acetabular parameters were significantly associated with the femoral head shape 5 years after diagnosis.

**Conclusion:**

The acetabulum developed an increasingly dysplastic shape in the course of Perthes’ disease. Early dysplastic changes of the acetabulum were not associated with a poor radiological outcome 5 years after diagnosis. Routine measurement and monitoring of acetabular changes in plain radiographs were of little prognostic value and can, therefore, hardly be recommended in clinical practice.

## Introduction

Perthes’ disease leads to typical anatomic changes of the femoral head [1–3]. In 1950, Heyman and Herndon emphasised that radiographic alteration of the acetabulum plays a crucial role in the evolution of the disease and they defined it as one of four major radiographic criteria to describe this condition [4]. In the last few decades, several authors have reported on radiographic changes of the acetabular anatomy, such as hypertrophy, retroversion, bicompartmental and dysplastic development [5–7]. It is still unknown as to whether these changes occur primarily or if they are secondary to the anatomical changes of the femoral head. The aim of our study was to describe radiographic changes of the acetabulum during the course of the disease and to assess whether early acetabular changes were associated with femoral head sphericity 5 years after diagnosis.

## Patients and methods

As part of the Norwegian prospective multi-centre study on Perthes’ disease, 425 patients were registered between 1996 and 2000 [8]. We analysed both the affected as well as the unaffected hips in all cases with unilateral involvement, age at onset 6 years or older and femoral head necrosis more than 50 % (*n* = 152). Radiographs were taken at diagnosis and at 1- and 5-year follow-up. The degree of femoral head necrosis was assessed according to the original Catterall classification [9]. We included radiographs classified as groups III and IV. Twenty-nine children were excluded due to inadequate exposure of the acetabular landmarks. Thus, 123 children (90 boys and 33 girls) with a mean age at the time of diagnosis of 7.5 years (range 6–13 years) were studied.

The radiographic phase was determined at the time of diagnosis according to Waldenström [10]. Sixty-three hips were in the initial phase (51 %), 48 were in the fragmentation phase (39 %), five were in the reossification phase (4 %) and seven hips had not been classified (6 %).

We applied the original lateral pillar classification of Herring et al. [11] in 110 patients at the fragmentation phase. Sixty hips were classified as lateral pillar type B (54.5 %) and 50 hips as lateral pillar type C (45.5 %). The femoral head cover was calculated as the percentage of the femoral head medial to Perkin’s line compared to the width of the femoral head, both measured parallel to Hilgenreiner’s line [4].

The children included in this study received either physiotherapy (*n* = 55), Scottish Rite orthosis (*n* = 26) or proximal femoral varus osteotomy (*n* = 71) [8], according to the choice of the local orthopaedic surgeons. The decision was based on surgeons’ preferences, treatment philosophy and local tradition. We combined patients treated with physiotherapy and orthosis into a non-operative treatment group.

Three different radiographic measurements were examined on antero-posterior (AP) pelvic radiographs to assess the acetabular anatomy, described in the following sections.

### Sharp’s angle

**Fig. 1:**
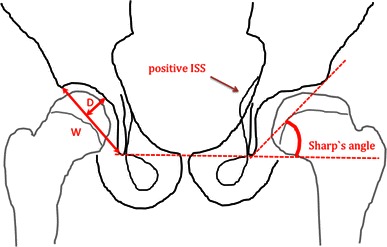
Drawing of an antero-posterior (AP) radiograph of the pelvis showing the left hip with Perthes’ disease and the right hip unaffected. Sharp’s angle is illustrated on the left hip. *W* acetabular width, *D* acetabular depth. The acetabular depth-to-width ratio (ADR) is defined as *D*/*W* × 1,000

This angle was described by Sharp in 1961 for the assessment of hip dysplasia [12]. A reference line was drawn between the inferior points of the teardrops on AP pelvis radiographs. The angle was formed by this reference line and a line connecting the inferior point of the teardrop and the lateral edge of the acetabulum (Fig. 1).

### Acetabular depth-to-width ratio (ADR)

The acetabular depth and width were measured on AP pelvic radiographs, as described by Heyman and Herndon in 1950 [4]. The length of a line connecting the lateral osseous acetabular margin and the lower end of the teardrop defined the width of the acetabulum. This teardrop is often more accurately defined than the lower acetabular margin. The depth was defined as the distance from the width line to the deepest point of the acetabulum (Fig. 1).

According to Cooperman et al. [13], we measured the ADR as depth/width × 1,000. We compared our results of the affected and unaffected hips with the depth-to-width quotients of 600 skeletally immature normal hips published by Bellemans et al. [14]. They established a normal range of the depth-to-width quotient for girls and boys. For the present study, we used Bellemans et al.’s results for children aged 6 years and older (Fig. 5). Values below 2 standard deviations (SDs) of the mean depth-to-width range for 6-year-olds were classified as dysplastically altered (boys: ADR <261; girls: ADR <274). We chose an ADR cut-off value of 265 based on the mean ADR between boys and girls.

### Lateral acetabular inclination

**Fig. 2:**
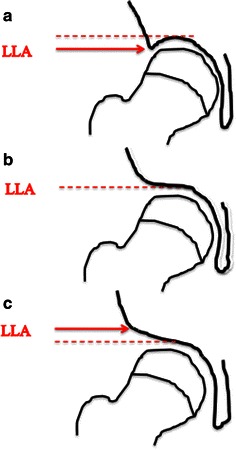
Schematic illustration of the lateral acetabular inclination. The lateral lip of the acetabulum (*LLA*) is (**a**) below, (**b**) horizontal or (**c**) above the weight-bearing dome (*dashed line*)

The lateral acetabular inclination was introduced by Cooperman et al. [13] in 1983 and later applied by Grzegorzewski et al. [15] for children with Perthes’ disease. It was recorded as down, horizontal or up, depending on whether the lateral lip of the acetabulum was below, horizontal or above the weight-bearing dome of the acetabulum, respectively (Fig. 2).

### Femoral head sphericity

Five years after diagnosis, hips were classified according the modified Stulberg three-group classification [16], where group A hips (Stulberg groups I and II) have a spherical head, group B hips (Stulberg group III) have an ovoid femoral head and group C hips (Stulberg groups IV and V) have a flat femoral head.

### Statistical analysis

We used IBM SPSS Statistics, version 20 for all statistical analyses. Numerical data were described as the mean and range. The mean differences between the affected and unaffected hips of patient groups were statistically compared with a paired-samples *t*-test. Categorical data were described using the number of observations (percentages) and analysed with cross-table analysis and Pearson’s Chi-squared tests.

An ordinal regression model was used to estimate the cumulative odds ratio (OR) for worse radiological outcome (modified Stulberg classification) with selected clinical predictor variables. The following variables were each analysed separately in an ordinal regression model: age at onset of the disease, gender, Catterall classification, lateral pillar classification, received treatment, femoral head coverage at 1-year follow-up and ADR. Further, those that were significantly associated with the Stulberg classification (with *p* ≤ 0.05 as criteria) were included in the final multi-variable ordinal regression model to assess their predictive ability regarding worse radiological outcome. Results were regarded as statistically significant if the *p*-values were below 0.05.

## Results

### Sharp’s angle

The mean value of the Sharp’s angle for the affected hip remained stationary throughout the course of the disease (Table 1). It decreased significantly in unaffected hips from diagnosis to the 5-year follow-up (*p* < 0.001). The difference between the Sharp’s angles of the affected and unaffected hips at the 5-year follow-up was significant (*p* < 0.001) (Table 1).

**Table 1:** Sharp’s angle and ADR values at the time of diagnosis and 1- and 5-year follow-up

Time of observation	Mean Sharp’s angle (SD)		Mean ADR (SD)	
	Perthes’ hip	Unaffected hip	Perthes’ hip	Unaffected hip
At diagnosis	45.5 (3.8)	44.9 (3.6) (*n* = 117)	282.3 (35.1)	305.1 (31.2) (*n* = 117)
1-year follow-up	45.0 (4.4)	44.4 (3.7) (*n* = 113)	254.9 (38.7)	310.2 (32.1) (*n* = 113)
5-year follow-up	45.7 (4.4)	42.2 (3.7) (*n* = 107)	263.3 (42.6)	309.1 (38.1) (*n* = 107)

*SD* standard deviation, *ADR* acetabular depth-to-width ratio, *n* number of patients; if the numbers are not specified in the table, it includes all 123 patients

### Acetabular depth-to-width ratio

**Fig. 3:**
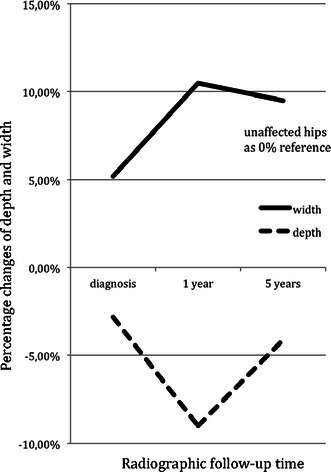
Figure showing changes of the acetabular depth and width from the time of diagnosis to 5-year follow-up (*x*-axis). The *y*-axis shows the percentage change of the depth (*dashed line*) and width (*solid line*) relative to the unaffected acetabula (0 % reference)

The mean ADR value of the affected hips at the time of diagnosis was significantly lower compared with the unaffected side (*p* < 0.001) (Table 1). This was due to both an increase of acetabular width (*p* < 0.001) and a slight decrease of depth (*p* < 0.001) (Fig. 3). We analysed the ADR at diagnosis separately according to each Waldenström radiographic phase. The mean ADR value of the affected hips in the initial phase was significantly lower compared with the unaffected side (287 vs. 302; *p* < 0.001).

The ADR of the affected hips decreased significantly from time of diagnosis to the 1-year follow-up (*p* < 0.001) (Table 1), with both a significant increase of acetabular width (*p* < 0.001) as well as depth (*p* < 0.001) (Fig. 3). At the 5-year follow-up, the mean ADR had increased significantly from the 1-year follow-up (*p* = 0.020). This partial normalisation was due to a stable acetabular width and a significant increase of the acetabular depth (*p* = 0.003) (Fig. 3). However, the ADR values were significantly lower in the affected hips at the 1- and 5-year follow-up compared with those of the unaffected hips (*p* < 0.001). In unaffected hips, the ADR did not change significantly during the course of the disease (Table 1).

### Lateral acetabular inclination

The proportion of affected hips with the lateral lip of the acetabulum below the weight-bearing dome decreased from the time of diagnosis to the 5-year follow-up, and the percentage of affected hips with an upward-sloping lateral margin increased during the course of the disease (Table 2). In the unaffected hips, there was a larger proportion of hips with the lateral lip below the weight-bearing dome at the time of diagnosis (53.0 %) (Table 2). This proportion increased during the course of the disease to 68.0 % at the 5-year follow-up. At diagnosis and 1-year follow-up, none of the unaffected hips had the lateral lip above the weight-bearing dome. This was also the case 5 years after diagnosis, except for one hip.

**Table 2:** Distribution of hips at the time of diagnosis and 1- and 5-year follow-up in relation to the lateral acetabular inclination

Lateral acetabular inclination	At diagnosis		1-year follow-up		5-year follow-up	
	Perthes’ hip	Unaffected hip	Perthes’ hip	Unaffected hip	Perthes’ hip	Unaffected hip
Down	36	62	11	70	3	83
Horizontal	78	55	78	43	60	38
Up	9	0	34	0	60	1
Total	123	117	123	113	123	122

The lateral acetabular inclination is classified as down, horizontal or up depending on whether the lateral lip of the acetabulum is above, at the same level or below the level of the weight-bearing dome

### Prognostic factors for a spherical femoral head at the 5-year follow-up

**Fig. 4:**
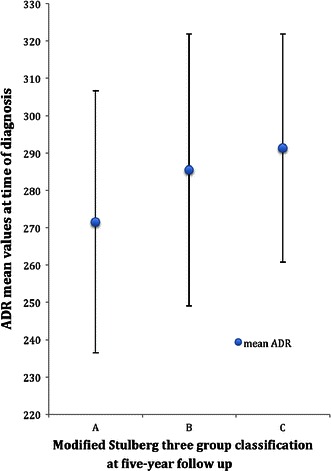
Diagram showing the relation between the mean ADR mean values at the time of diagnosis and the modified Stulberg classification at the 5-year follow-up

For the evaluation of factors that might influence the radiological outcome at the 5-year follow-up, we performed an ordinal regression analysis for a single variable for each of the following parameters: Sharp’s angle, lateral acetabular inclination, ADR, age at diagnosis, gender, Catterall classification, lateral pillar classification, treatment and femoral head coverage. The results are given in Table 3. Of the three acetabular measurements, only the ADR was significantly associated with the modified Stulberg classification at the 5-year follow-up. Lower ADR values at the time of diagnosis were associated with a spherical femoral head (group A) and higher ADR values with a flat femoral head (group C) (Fig. 4). Using the ADR cut-off value of 265 at the time of diagnosis, we grouped the affected hips into ‘normal’ (ADR >265, *n* = 84) and ‘wider-shallower hips’ (ADR <265, *n* = 39). Patients with normal acetabula at diagnosis developed a flat femoral head in 34 % of the cases, while patients with wider and shallower acetabula developed a flat femoral head in 8 % of the cases in the evolution of Perthes’ disease. In order to assess the predictive ability for femoral head sphericity at the 5-year follow-up, we performed a final multi-variable ordinal regression model including all parameters with significant association in the single-variable analysis. The results show that treatment, lateral pillar classification and Catterall classification were strongly associated with the femoral head sphericity (Table 4). The ADR at diagnosis showed a trend but the association was not statistically significant (*p* = 0.061).

**Table 3:** Simple ordinal regression analysis, i.e. separate analysis for each prognostic factor, with three-group Stulberg classification as the outcome variable

Prognostic factor	OR	95 % CI	*p*-Value
Lateral pillar classification	0.188	0.086–0.410	<0.001
Treatment	2.731	1.384–5.391	0.004
Catterall classification	0.156	0.073–0.332	<0.001
ADR at diagnosis	1.012	1.002–1.022	0.016
Femoral head coverage at 1 year	0.971	0.942–1.002	0.064
Age at diagnosis	1.012	0.988–1.035	0.334
Gender	1.213	0.566–2.600	0.620
Sharp’s angle	1.021	0.036–1.114	0.641
Acetabular lateral inclination^a^	0.792	0.219–2.858	0.721
	1.558	0.731–3.323	0.251

*OR* odds ratio, *95* *% CI* 95 % confidence interval, *ADR* acetabular depth-to-width ratio

^a^An ordinal regression analysis of a three-group categorical parameter with the Stulberg classification leads to two odds ratio results

**Table 4:** Multi-variable ordinal regression analysis with the modified three-group Stulberg classification as the outcome variable

Prognostic factor	OR	95 % CI	*p*-Value
Lateral pillar classification	0.276	0.119–0.644	0.003
Treatment	2.927	1.349–6.350	0.007
Catterall classification	0.324	0.138–0.760	0.010
ADR at diagnosis	1.011	1.000–1.022	0.061

*OR* odds ratio, *95* *% CI* 95 % confidence interval, *ADR* acetabular depth-to-width ratio

## Discussion

### Sharp’s angle

The unaffected acetabulum followed the normal age-dependent development of Sharp’s angle as reported by Ozçelik et al. [17], whereas the affected side remained steep at the 5-year-follow-up. One previous study has measured the Sharp’s angle in children with Perthes’ disease. Joseph showed that the Sharp’s angle of Perthes’ hips in male, skeletally immature patients was higher compared to the unaffected side (44.7° vs. 42.9°) [5]. Furthermore, he found a significant difference in the Sharp’s angle between affected and unaffected hips in symptomatic adult patients with healed Perthes’ disease (40.1° and 36.1°, respectively). These results are in accordance with our results, indicating that a steeper acetabulum is developed in Perthes’ hips during the course of the disease.

### Acetabular depth-to-width ratio

Heyman and Herndon showed that acetabulum on the affected side developed increased width and decreased depth in children with Perthes’ disease [4]. The present study identified when these changes occurred during the course of the disease. We found an increase in acetabular width within the first year of the disease, but no further increase until the 5-year follow-up. After initial decrease, the acetabular depth normalised during the course of the disease, indicating that the acetabulum was remodelling to an anatomically more normal shape. However, affected acetabula were wider and shallower at all stages of the disease compared with the unaffected side.

**Fig. 5:**
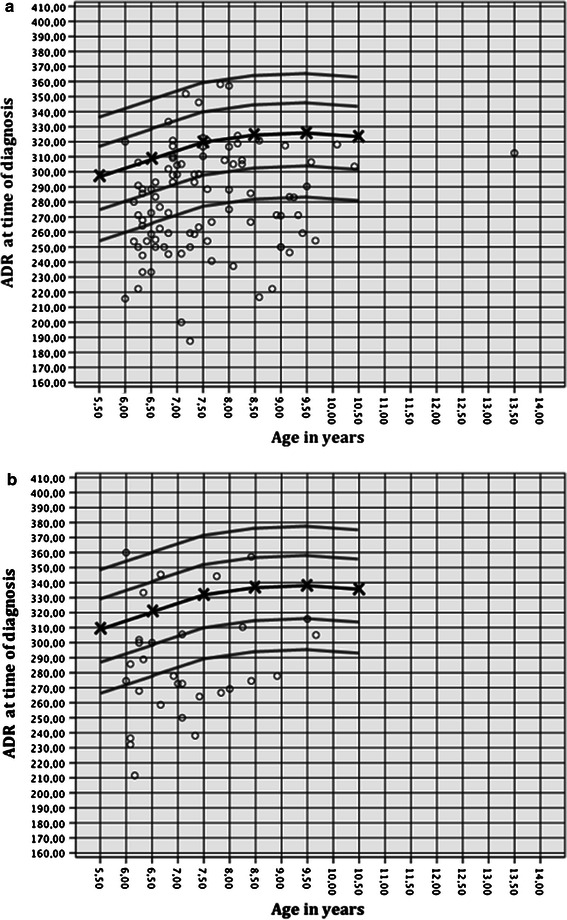
ADR at the time of diagnosis for (**a**) boys and (**b**) girls in relation to age, as published by Bellemans et al. [14] (mean, 1 SD and 2 SD)

**Fig. 6:**
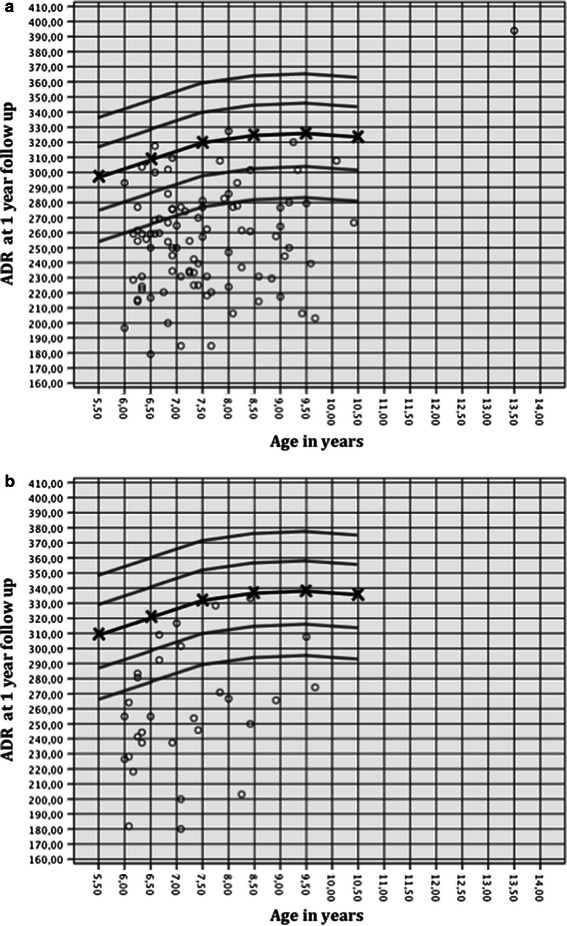
ADR at the 1-year follow-up for (**a**) boys and (**b**) girls in relation to age, as published by Bellemans et al. [14] (mean, 1 SD and 2 SD)

Bellemans et al. [14] showed that the ADR values increased from a base level of approximately 250 at 3 years of age to values over 300 at the age of 8 years and older in 600 normal hips. We compared the ADR values of the affected side at time the of diagnosis with the ADR values reported by Bellemans et al. and found that a majority was below the normal range for both girls and boys (Fig. 5). This tendency was aggravated at the 1-year follow-up (Fig. 6). These findings indicate early dysplastic alteration of the acetabulum in terms of increased width and decreased depth, as reported by other authors [5, 14].

### Lateral acetabular inclination

Grzegorzewski et al. [15] evaluated 243 cases of unilateral Perthes’ disease at fragmentation and at skeletal maturity. They found that the lateral acetabular margin of the affected hips was below the weight-bearing dome of the acetabulum in 32 %, horizontal in 56 % and above in 12 % at fragmentation. These results are in accordance with our observations for affected hips at the time of diagnosis (Table 2). There was a consistent increase of affected acetabula with an upward-sloping lateral margin until the 5-year follow-up, which is markedly higher than that reported by Grzegorzewski et al. [15]. However, direct comparison is difficult because of differences between the study populations.

### Prognostic factors for a spherical femoral head

To our knowledge, no previous study has associated Sharp’s angle at an early stage of Perthes’ disease with the later shape of the femoral head. We found no significant associations between this parameter at the time of diagnosis and at the 1-year follow-up with the modified three-group Stulberg classification at the 5-year follow-up. Thus, Sharp’s angle is of no prognostic value in Perthes’ disease.

Grzegorzewski et al. [15] found an association between an upward-sloping lateral margin and a flat femoral head shape at skeletal maturity. They hypothesised that a deformed lateral margin could lead to decreased anatomical support for the femoral head and, thereby, cause further subluxation and flattening of the femoral head. Our results do not support these findings, as we found no significant association between the lateral acetabular inclination early in the course and the femoral head shape 5 years after diagnosis. Therefore, routine measurement and monitoring of Sharp’s angle and lateral acetabular inclination can hardly be recommended in clinical practice.

As previous studies on this subject have shown, we found that lateral pillar height of more than 50 %, operative treatment and femoral head necrosis less than 75 % were the three strongest predictors for a spherical femoral head [8, 18]. Bellemans et al. [14] stated that a dysplastic acetabulum was associated with poor radiographic outcome in Perthes’ disease. This is not in accordance with our results, as we found that wider and shallower acetabula at diagnosis were associated with a more favourable late radiographic outcome. However, the ADR had no significant prognostic value in the multi-variable regression test and, therefore, has limited value in clinical practice.

### Development over time and possible causes of acetabular changes in Perthes’ hips

Perthes’ disease leads to an enlargement and lateralisation of the femoral head, thereby altering the force transmission from the femoral head to the acetabulum. Madan et al. [19] observed that the acetabulum became shallower and steeper secondary to the abnormal shape and size of the femoral head. They stated that the alteration in acetabular growth was due to lateral pressure from the deforming femoral head. Our results support only to some extent the theory of mechanically induced alterations of the acetabulum, since a wider and shallower acetabulum was already present at diagnosis in all hips, including those that were in the initial radiographic phase. These changes were most likely not caused by mechanical forces alone.

Acetabular development occurs by a combination of enchondral, interstitial and appositional growth [20]. Our observations support the work of Joseph. He suggested that some of the early acetabular changes do not necessarily follow the alterations of the shape of the femoral head [5]. He and others showed significantly increased metabolic activity in the area of the triradiate cartilage of the affected hip at an early stage [5, 19]. They hypothesised that hyperaemia may lead to increased growth at the triradiate cartilage, causing a widening of the acetabulum. Growth alteration due to hyperaemia in close relation to the growth plate is a phenomenon also known in other conditions, such as juvenile idiopathic arthritis, where higher metabolic activity may cause excessive growth [21]. Several authors found a thickening of medial cartilage of the acetabulum due to inflammatory processes and increased metabolism [6, 22, 23], leading to hypertrophy and reduced acetabular depth, independent of mechanical forces. Joseph suggested that increased appositional growth was mediated by synovitis and hyperaemia, being a factor contributing to depth reduction [5].

In summary, our results suggest that the dysplastic changes of the acetabulum in Perthes’ disease are evoked by primary and secondary mechanisms. Primarily, the disease induces excessive growth, causing widening and hypertrophy of the acetabulum that might be induced by hyperaemia and higher metabolic activity. Secondarily, altered dimensions of the femoral head lead to mechanically induced changes, which inhibit the natural tilt of the acetabulum and cause an upward-sloping lateral margin. Early dysplastic changes of the acetabulum were not associated with a poor radiological outcome 5 years after diagnosis. Contrarily, there was a tendency that children with a wider and shallower acetabulum had a lesser degree of later femoral head deformation.
